# Inhibition of calpain reduces cell apoptosis by suppressing mitochondrial fission in acute viral myocarditis

**DOI:** 10.1007/s10565-021-09634-9

**Published:** 2021-08-08

**Authors:** Hui Shi, Ying Yu, Xiaoxiao Liu, Yong Yu, Minghui Li, Yucheng Wang, Yunzeng Zou, Ruizhen Chen, Junbo Ge

**Affiliations:** 1 Department of Cardiology, Shanghai Institute of Cardiovascular Diseases, Zhongshan Hospital, Shanghai Medical College of Fudan University, Shanghai, 200030 China; 2 Department of General Practice, Zhongshan Hospital, Shanghai Medical College of Fudan University, Shanghai, 200030 China

**Keywords:** Calpain, Apoptosis, Mitochondria, Dynamin-related protein 1, Viral myocarditis

## Abstract

Cardiomyocyte apoptosis is critical for the development of viral myocarditis (VMC), which is one of the leading causes of cardiac sudden death in young adults. Our previous studies have demonstrated that elevated calpain activity is involved in the pathogenesis of VMC. This study aimed to further explore the underlying mechanisms. Neonatal rat cardiomyocytes (NRCMs) and transgenic mice overexpressing calpastatin were infected with coxsackievirus B3 (CVB3) to establish a VMC model. Apoptosis was detected with flow cytometry, TUNEL staining, and western blotting. Cardiac function was measured using echocardiography. Mitochondrial function was measured using ATP assays, JC-1, and MitoSOX. Mitochondrial morphology was observed using MitoTracker staining and transmission electron microscopy. Colocalization of dynamin-related protein 1 (Drp-1) in mitochondria was examined using immunofluorescence. Phosphorylation levels of Drp-1 at Ser637 site were determined using western blotting analysis. We found that CVB3 infection impaired mitochondrial function as evidenced by increased mitochondrial ROS production, decreased ATP production and mitochondrial membrane potential, induced myocardial apoptosis and damage, and decreased myocardial function. These effects of CVB3 infection were attenuated by inhibition of calpain both by PD150606 treatment and calpastatin overexpression. Furthermore, CVB3-induced mitochondrial dysfunction was associated with the accumulation of Drp-1 in the outer membrane of mitochondria and subsequent increase in mitochondrial fission. Mechanistically, calpain cleaved and activated calcineurin A, which dephosphorylated Drp-1 at Ser637 site and promoted its accumulation in the mitochondria, leading to mitochondrial fission and dysfunction. In summary, calpain inhibition attenuated CVB3-induced myocarditis by reducing mitochondrial fission, thereby inhibiting cardiomyocyte apoptosis.

**Figure:**
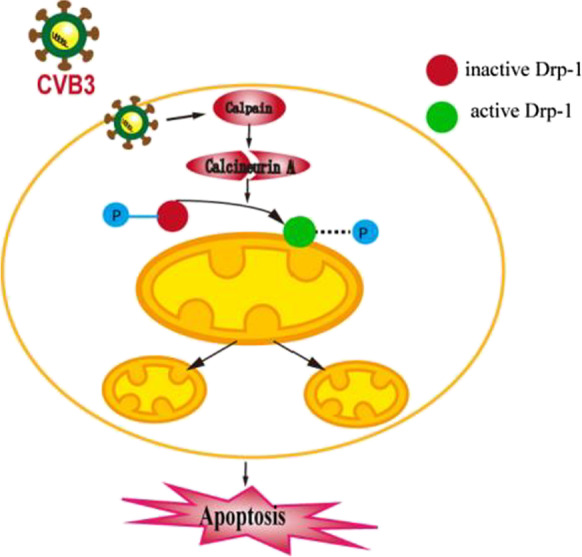
Graphical abstract Calpain is activated by CVB3 infection. Activated calpain cleaves calcineurin A and converts it to active form which could dephosphorylate Drp-1 at Ser637 site. Then, the active Drp-1 translocates from the cytoplasm to mitochondria and triggers excessive mitochondrial fission. Eventually, the balance of mitochondrial dynamics is broken, and apoptosis occurs.

Calpain is activated by CVB3 infection. Activated calpain cleaves calcineurin A and converts it to active form which could dephosphorylate Drp-1 at Ser637 site. Then, the active Drp-1 translocates from the cytoplasm to mitochondria and triggers excessive mitochondrial fission. Eventually, the balance of mitochondrial dynamics is broken, and apoptosis occurs.

## Introduction

Viral myocarditis (VMC) which can progress to dilated cardiomyopathy is identified as a principal cause of sudden death in young adults (Bejiqi et al. 2019). Although there have been numerous studies, there are still challenges to discovering effective ways to treat this difficult disease. Coxsackievirus B3 (CVB3) is the most common and most extensively studied pathogen in human and animal models. Although the pathogenesis of VMC induced by CVB3 is not fully understood, many research have revealed that apoptosis is one of the important pathological changes in VMC. Cardiomyocyte apoptosis caused by host interferon responses or release of progeny virus contributes to myocyte loss in the acute phase of VMC (Corsten et al. 2012). Additionally, plenty of evidence have indicated the protective role of inhibiting apoptosis in VMC (Li et al. 2019; Liu et al. 2019). Thus, it is essential to figure out the underlying mechanisms of myocyte apoptosis induced by CVB3 and attempt to reduce CVB3-mediated apoptosis.

Mitochondrial function is essential for cardiomyocytes because mitochondria not only constantly generate ATP to maintain normal heart function but are also crucial for regulating reactive oxygen species (ROS) production, calcium homeostasis, and apoptosis (Marin-Garcia and Akhmedov 2016). Given the high density of mitochondria in cardiomyocytes and the dependence of cardiomyocytes on mitochondria, it is reasonable to assume that pathological alterations in the heart are related with changes in mitochondrial function (Huss and Kelly 2005). In the VMC model, numerous changes in mitochondrial function have been reported, including mtDNA deletion, loss of mitochondrial membrane phospholipids (Wei et al. 2014), decreased respiratory chain complex activities, and increased ROS production (Ebermann et al. 2012). Consequently, mitochondria may be a target during viral infection.

Mitochondria are highly dynamic organelles undergoing persistent fusion-fission processes that are controlled by a number of proteins. Equilibrium between these two opposing processes is required for maintaining proper mitochondrial function (Ahmed et al. 2019). Excessive fission leads to abnormal mitochondrial fragmentation, which causes mitochondrial dysfunction including defective cellular metabolism, ROS overproduction, and apoptosis (Ahmed et al. 2019). The major protein that regulates mitochondrial fission is dynamin-related protein 1 (Drp-1). Primarily located in the cytoplasm, Drp-1 transfers from the cytoplasm to the outer membrane of mitochondria (OMM) where it interacts with its receptors to initiate mitochondrial fission (Ong et al. 2015). Recently, excessive mitochondrial fission mediated by Drp-1 has been involved in many cardiovascular diseases and is considered as a potential therapeutic target (Ong et al. 2015; Zu et al. 2020). Moreover, viruses such as hepatitis C virus could induce mitochondrial fission by stimulating the expression of Drp-1 to facilitate viral infection and persistence (Kim et al. 2014). Furthermore, Lin et al. indicated that CVB3 infection caused the perturbation of mitochondrial dynamics by promoting the migration of Drp-1 from the cytoplasm to mitochondria, which causes apoptosis and inflammation in VMC (Lin et al. 2017). As a result, Drp-1 may serve as a promising target for treating cardiovascular diseases, including VMC.

Calpain, an intracellular Ca^2+^-dependent cysteine protease, is ubiquitously distributed in many tissues and associated with many important cellular pathophysiological processes. Calpain can facilitate apoptosis by proteolyzing various substrates that can promote cells to apoptosis (Storr et al. 2011). Activation of calpains has involved in many cardiac diseases, and pharmacological and genetic inhibition of calpains protects the heart from cardiac injury (Letavernier et al. 2012; Poncelas et al. 2017; Li et al. 2020). Additionally, our previous work demonstrated increased activity of calpain in VMC, implying that calpain plays pivotal roles in VMC (Li et al. 2016). However, the underlying mechanism is still unclear. The recent study revealed that upregulated calpain activity could result in mitochondrial fragmentation and neuronal apoptosis (Takano et al. 2005; Tangmansakulchai et al. 2016). Therefore, we hypothesized that calpain activation induces mitochondrial fragmentation by activating Drp-1, which causes cardiomyocyte apoptosis in VMC.

## Materials and methods

### Cell culture and treatment

PD150606 (Abcam, MA, USA), Mdivi-1 (MCE, Shanghai, China), and FK506 (MCE, Shanghai, China) were used as the inhibitors of calpains, Drp-1, and calcineurin A, respectively. Neonatal rat cardiomyocytes (NRCMs) were isolated from Neonatal Sprague–Dawley (SD) rats (1–3-day-old) as previously described (Jiang et al. 2019). Cardiomyocytes were cultured in DMEM (HyClone, South Logan, USA) supplemented with 10% fetal bovine serum (Gibco, NY, USA). 5-Bromodeoxyuridine (5-BrdU, 0.1 mM) (Sigma-Aldrich, MO, USA) was used to inhibit fibroblast proliferation.

NRCMs were infected with CVB3 (Nancy strain), which had been used as previously described (Li et al. 2016). The cells were divided into following groups: control group, PD150606 group, virus group, and virus + PD150606 group, Mdivi-1 group, virus + Mdivi-1 group, FK506 group, and virus + FK506 group.

### Flow cytometric analysis for cell apoptosis

Cardiomyocyte apoptosis was quantified by annexin V/PI apoptosis assay (BD, CA, USA). Briefly, harvested cells were rinsed with cold PBS and resuspended in binding buffer. Annexin V and PI were added to 100 μl of cell suspension. After incubating at room temperature for 15 min protected from light, 400 μl binding buffer was added to each tube. The analysis was conducted using a fluorescence-activated cell sorting machine with FlowJo software.

### Mitochondrial membrane potential measurement

Mitochondrial membrane potential (MMP) was monitored by using JC-1staining kit (Beyotime, Shanghai, China). Cells were stained by JC-1 (10 μg/mL) at 37 °C for 20 min. MMP was visualized by fluorescence microscopy (Olympus, Tokyo, Japan). JC-1 monomers appear green fluorescence, indicating low MMP. JC-1 aggregates present red fluorescence, suggesting high MMP. Data are represented as the ratio of red to green fluorescence intensity.

### ATP assay

Enhanced ATP assay kit was used to measure ATP contents (Beyotime, Shanghai, China). NRCMs and tissues were lysed in ATP lysis buffer and then were centrifuged at 12,000 rpm for 10 min to acquire supernatant. The supernatant and ATP detection working dilution were mixed. The ATP contents were measured by a microplate luminometer, and the amount was normalized to the protein concentration.

### Mitochondrial ROS measurement

Detection of mitochondrial ROS was conducted by MitoSOX Red according to the manufacturer’s instruction (Invitrogen, CA, USA). Briefly, 5 mM stock solution was prepared by using DMSO. Then, samples were incubated with working solution (5 μM) for 10 min at 37 °C in the dark. Lastly, cells were observed by a fluorescence microscope (Olympus, Tokyo, Japan).

### Mitochondrial imaging

NRCMs were incubated with 200 nM MitoTracker Green (Beyotime, Shanghai, China) in a medium at 37 °C. After 30 min, the dye was replaced with complete medium. Mitochondrial morphology was observed by laser confocal microscopy (Leica, Wetzlar, Germany).

### Immunofluorescence

Fixed samples were permeabilized with 0.5% Triton X-100 for 15 min. Samples were blocked with 5% BSA for 1 h and incubated with primary antibodies, Drp-1 (1:50, Cell Signaling Technology, MA, USA) and COX-IV (1:100, Cell Signaling Technology, MA, USA), overnight at 4 °C. Then, the cells were washed with PBS and incubated with the corresponding Alexa-labeled secondary antibodies (Invitrogen, CA, USA). Finally, samples were stained with DAPI and observed with a confocal microscope (Leica, Wetzlar, Germany).

### Animals

The transgenic mice overexpressing calpastatin (Tg-CAST) were acquired from Dr. Tianqing Peng’s laboratory (Lawson Health Research Institute, Canada) and bred in a standard specific pathogen-free (SPF) environment in Fudan University. Transgenic mice and littermate wild-type (WT) mice aged 3–4 weeks were divided into four groups: WT control group (*n* = 8), Tg-CAST control group (*n* = 8), CVB3-infected group (*n* = 8), and CVB3-infected Tg-CAST group (*n* = 8). The myocarditis animal model was established by using CVB3. Briefly, 100,000 TCID_50_ × 0.1 ml CVB3 was injected intraperitoneally into mice. After 7 days, the mice were anesthetized using 1.5% pentobarbital. Heart tissues were harvested. Blood samples were obtained and centrifuged at 3000 r/min for 10 min to acquire serum. The experimental procedures were conducted with prior approval by the ethical committee of Fudan University.

### Viral titration

Heart tissues were homogenized to acquire virus-containing supernatant. Proportionally dilutions of tissue supernatant were added to Hela cells. Hela cells were used to measure the virus titer by TCID_50_ as described before (Li et al. 2016).

#### ELISA

Serum was collected as described above. The levels of CK-MB and cTnI in the peripheral blood were determined by commercially available ELISA kits according to the manufacturer’s instructions (Haoben, China).

### Histopathology, ROS detection, and TUNEL assay

Fixed heart tissues were stained with hematoxylin and eosin (H&E). Immunohistochemistry was performed as previously described (Li et al. 2016). A TUNEL assay (Roche, Switzerland) was performed to determine apoptosis in heart tissues according to the manufacturer’s instructions. For ROS detection, 4-µm frozen sections were treated with DHE at a concentration of 50 µM for 7 min in the dark and then were washed with PBS. Stained sections were observed using a fluorescence microscope (Olympus, Tokyo, Japan). Six fields were randomly selected for observation. All images were examined in a blinded manner.

### Echocardiography

All mice were anesthetized with isoflurane, and echocardiographic measurements were conducted using an echocardiography system (Vevo Visualsonics 2100, Canada). M-mode parameters were obtained to calculate left ventricular ejection fraction (LVEF) and LV fractional shortening (FS).

### Transmission electron microscopy (TEM)

Small pieces (1 mm^3^) of fresh heart tissue were fixed with 2.5% glutaraldehyde. The blocks were then postfixed in 1% OsO4, followed by dehydration in ethanol. Tissues were infiltrated, embedded, and then sliced to acquire ultrathin sections by ultramicrotome. Finally, transmission electron microscopy was used to observe mitochondrial morphology in stained sections with uranyl acetate and leas citrate (Hitachi, Tokyo, Japan).

### Quantitative real-time polymerase chain reaction (PCR)

Total RNA extraction was performed by using Trizol Reagent (Invitrogen, USA). Then the RNA was reverse transcribed into c-DNA using Transcript PCR kit (TaKaRa, Japan). Quantitative real-time PCR using the TB GreenTM Fast qPCR Mix (TaKaRa, Japan) was performed in a Bio-Rad CFX Connect™ real-time detection system. Primer sequences of qPCR were listed in Table 1.

**Table 1:** A list of qPCR primers

	Forward	Reverse
Drp-1	5’-GGTCATCAATAAGCTGCAAGAC-3’	5’-GTTCCCACTACGACAATCTGAG-3’
Fis-1	5’-AAAGACTCCAGCTGATAGATCG-3’	5’-GTCGTCATTGTATTTGCTTCGA-3’
Opa-1	5-CTTACATGCAGAATCCTAACGC-3’	5’-CCAAGTCTGTAACAATACTGCG-3’
Mfn-1	5’-GAAGAAAAGCGTGAAGACTGTT-3’	5’-CCACCAAAACAAAAACATCAGC-3’
Mfn-2	5’-TACAATGATCAGGTTCAGCGTC-3’	5’-ATAGAGGTCCTGGAAGTCAGAG-3’
GAPDH	5’-AACGACCCCTTCATTGAC-3’	5’-TCCACGACATACTCAGCAC-3’

### Western blot analysis

Total proteins were collected using RIPA (Beyotime, Shanghai, China). In addition, the mitochondrial proteins and cytoplastic proteins were extracted using the commercial mitochondrial isolation kit according to the manufacturer’s protocol (Beyotime, Shanghai, China). Samples containing 20 μg proteins from each group were separated by SDS-PAGE gels and transferred to PVDF membranes. After blocking, the membranes were incubated with various primary antibodies at 4 °C overnight, including Bax (1: 2000, Proteintech, Manchester, UK), Bcl-2 (1: 250, Santa Cruz, CA, USA), cleaved-Caspase3 (1:1000, Cell Signaling Technology, MA, USA), VP-1 (1:1000, GeneTex, CA, USA), Drp-1 (1:1000, Cell Signaling Technology, MA, USA), p-Drp-1 (ser637) (1:1000, Affinity Biosciences, OH, USA), α-fodrin (1:1000, Enzo Life Sciences, Raamsdonksveer, Netherlands), COX-IV (1:1000, Cell Signaling Technology, MA, USA), calcineurin A (1:1000, Cell Signaling Technology, MA, USA), and GAPDH (1:4000, KangChen Biotech, China). The membranes were incubated with the corresponding HRP-conjugated secondary antibodies (1:8000, Jackson ImmunoResearch, PA, USA). Then, the membranes were visualized by enhanced chemiluminescence (Millipore, Billerica, MA, USA). The quantification of blot bands was analyzed by ImageJ software.

### Statistical analysis

Data are expressed as the mean ± SEM. Differences among multiple groups were determined using one-way ANOVA with the post hoc Bonferroni test. All data were analyzed using GraphPad Prism 7 (GraphPad Software Inc., CA, USA). *P* < 0.05 was considered statistically significant.

## Results

### Calpastatin overexpression ameliorated CVB3-induced myocardium injury and promoted cardiac function in VMC mice

**Fig. 1:**
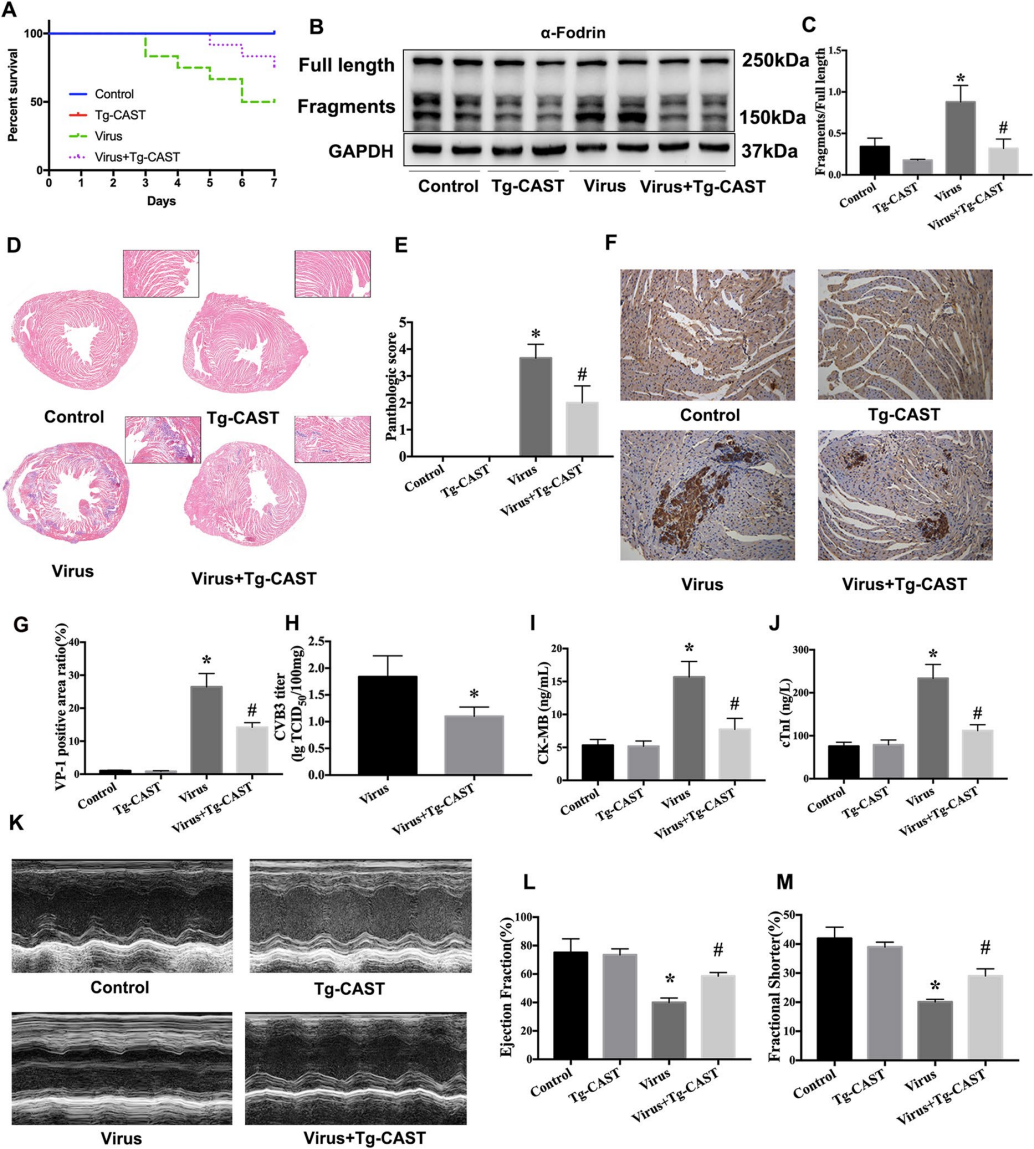
Calpastatin overexpression ameliorated CVB3-induced myocardium injury and promoted cardiac function in VMC mice. **a** Survival curve of the mouse in different groups. **b**, **c** Western bolts were used to evaluate the cleaved-α-fodrin in mice. **d** HE staining was performed to indicate inflammatory lesions in the heart tissues (× 100). **e** Quantitative analysis of pathological score. **f** Immunohistology staining of VP1 in heart tissue (× 400). **g** Quantitative analysis of positive area of VP-1. **h** Virus load titration in heart tissue. **i**, **j** ELISA assays were used to measure the level of peripheral myocardial damage markers, CK-MB (**i**), and cTnI (**j**). **k** M-mode echocardiography was used to evaluate the cardiac function of mice in each group. **l** The changes of left ventricular ejection fraction. **m** The changes of left ventricular fractional shortening. Control, littermate wild-type mice; Tg-CAST, calpastatin overexpression mice; virus, WT mice infected with CVB3; virus + Tg-CAST, Tg-CAST mice infected with CVB3. Data are expressed as mean ± SEM. *P < 0.05 vs. Con; #*P* < 0.05 vs. virus group

First, we found that the Tg-CAST + virus group had lower mortality than the virus group (Fig. 1a). Next, we examine the calpain activity in CVB3-infected mice. α-Fodrin could be cleaved by activated calpain, so the degradation of α-fodrin was examined to determine the role of calpain activation in CVB3-induced VMC mice (Li et al. 2016). We found that the ratio of cleaved fragments over intact α-fodrin was upregulated in the virus group, while calpastatin overexpression decreased this ratio induced by CVB3 infection in VMC mice (Fig. 1b, c). These findings suggest that calpain activity was upregulated due to CVB3 infection in vivo. Endogenous inhibition of calpain activity also mitigated inflammatory responses in heart tissue after viral infection (Fig. 1d, e). CVB3-infected Tg-CAST mice had lower virus titer and lower level of CVB3 capsid protein VP1 than those in the virus group (Fig. 1f–h). Furthermore, the markers of myocardial injury, CK-MB and cTnI, were elevated in the virus group, while the levels of these two makers were reduced in CVB3-infected transgenic mice, indicating significantly decreased myocardial injury (Fig. 1i, j). We also examined the effect of calpastatin overexpression on cardiac function following CVB3 infection. LVEF and LVFS were significantly reduced in the virus group. In contrast, calpastatin overexpression prevented CVB3-induced cardiac dysfunction (Fig. 1k–m). Taken together, these findings confirmed the protective role of inhibiting calpain activity in mice suffering from VMC.

### Calpastatin overexpression decreased myocardial apoptosis in VMC mice

**Fig. 2:**
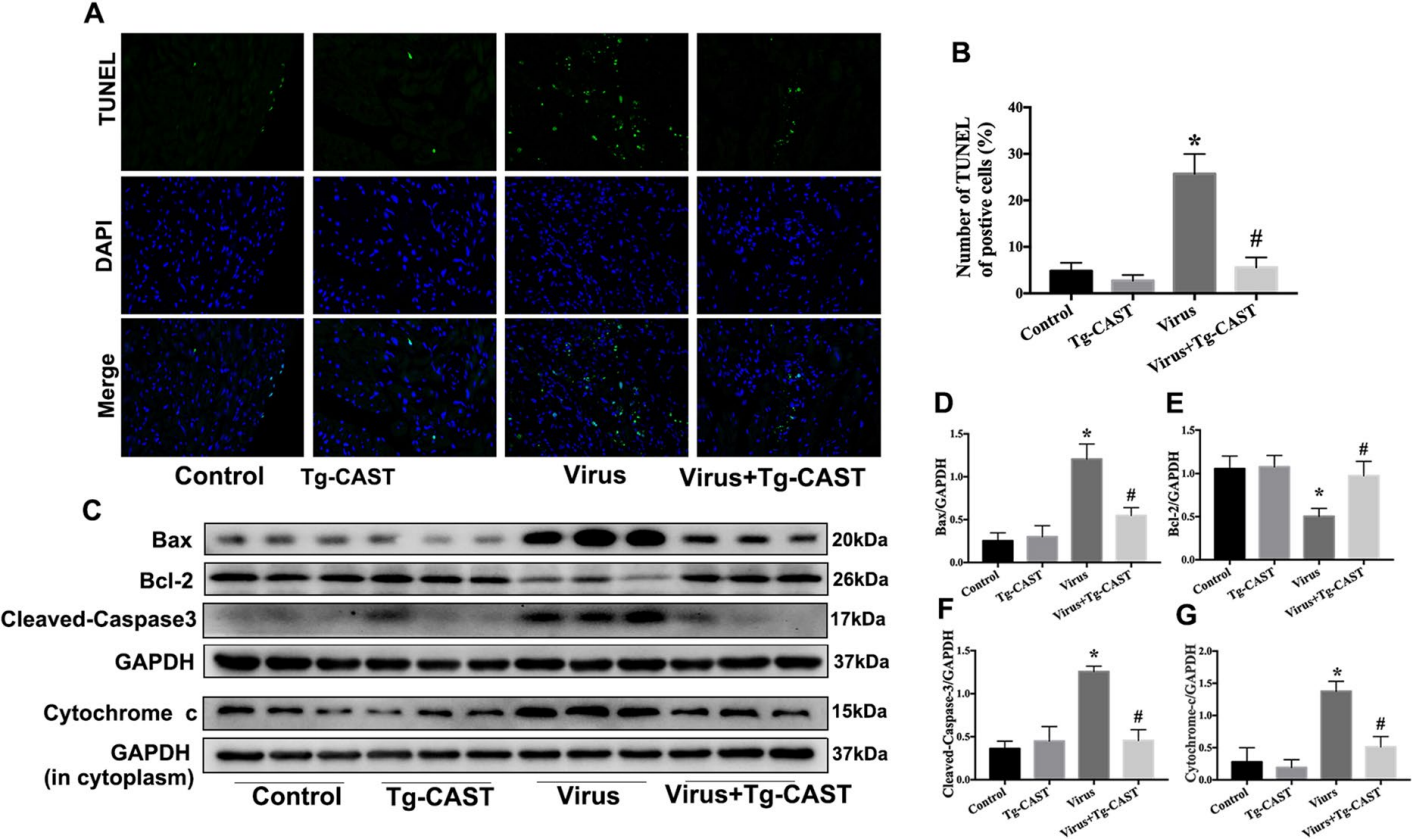
Calpastatin overexpression decreased apoptosis in myocardium in VMC mice. **a**, **b** TUNEL assay was used to quantify the cellular apoptosis in each group (× 400). TUNEL, green fluorescence presents TUNEL-positive nuclei; DAPI, blue fluorescence presents the total nuclei of cells. **c**–**g** Western bolts were used to measure the change of proteins related to apoptosis, Bax (**d**), Bcl-2 (**e**), cleaved-Caspase-3 (**f**), and cytochrome **c** in the cytoplasm (**g**). Data are expressed as mean ± SEM. *P < 0.05 vs. Con; #*P* < 0.05 vs. virus group

Virus group has more TUNEL-positive cells compared with that of the control and Tg-CAST groups. There was a decrease in the number of apoptotic cells in transgenic mice infected with CVB3 (Fig. 2a, b). The levels of cyt-c in the cytoplasm and the expression of Bax and cleaved-Caspase-3 were significantly increased, and Bcl-2 expression levels were decreased in the virus group. However, calpastatin overexpression inhibited apoptosis in CVB3-infected transgenic mice, manifested by the downregulation of Bax and cleaved-Caspase-3 and the upregulation of Bcl-2 (Fig. 2c–g). These results show that the overexpression of calpastatin attenuated apoptosis in the myocardium of mice caused by CVB3 infection.

### Calpastatin overexpression preserved mitochondrial function and prevented excessive mitochondrial fission via inhibition of Drp-1 translocation in VMC mice

**Fig. 3:**
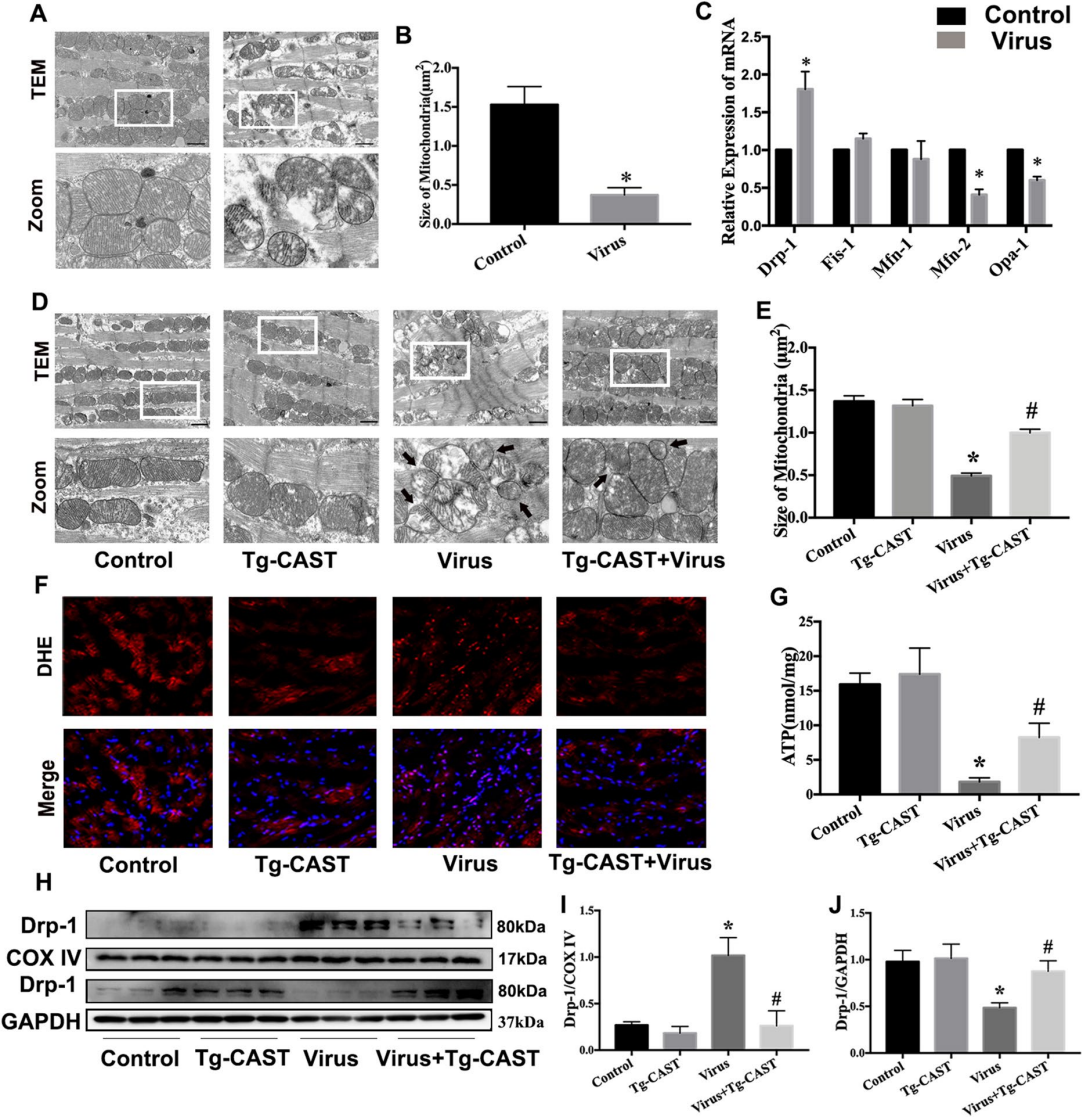
Calpastatin overexpression preserved mitochondrial function and prevented excessive mitochondrial fission via inhibition of Drp-1 translocation in VMC mice. **a** Mitochondrial morphology was observed by TEM in control and virus group. The second panel shows the magnified regions; scale bar = 1 μm. **b** The average size of mitochondria in control and virus group. **c** The mRNA levels of Drp-1, Fis-1, Mfn-1, Mfn-2, and Opa-1 in control and virus group. **d** Mitochondrial morphology was observed by TEM. Black arrow indicates fragmented mitochondria; scale bar = 1 μm. **e** The average size of mitochondria in each group. **f** Intracellular ROS levels were examined by DHE. **g** The results of ATP levels in each group. **h**–**j** The mitochondrial proteins and cytoplastic proteins were extracted from hearts of each group. Western bolts were used to evaluate Drp-1 expression in mitochondria (**i**) and cytoplasm (**j**), respectively, in each group. Data are expressed as mean ± SEM.*P < 0.05 vs. Con; #*P* < 0.05 vs. virus group

It has been reported that imbalanced mitochondria dynamic is associated with cell apoptosis (Lee et al. 2016). Therefore, we observe the mitochondrial ultrastructure by TEM. Mitochondrial structure was obviously damaged in the virus group, as manifested by irregular arrangement, disintegrated cristae, and excessive fission (Fig. 3a, b). We measured the mRNA levels related to mitochondria dynamic. CVB3 infection increased the mRNA levels of Drp-1 while decreasing the levels of Mfn-2 and Opa-1 in heart tissues (Fig. 3c). These findings suggest that imbalanced mitochondrial dynamic may participate the apoptosis in VMC mice.

Next, we used Tg-CAST mice to detect the relationship between calpain activation and mitochondrial excessive fission in VMC mice. Calpastatin overexpression ameliorated mitochondrial structure damage in mice infected with CVB3 (Fig. 3d, e). Our results also demonstrate that the overexpression of calpastatin reduced ROS production (Fig. 3f) and partly restored ATP synthesis in Tg-CAST mice infected with CVB3 (Fig. 3g).

According to the former results, we examined Drp-1 protein expression in each group. Since the translocation of Drp-1 activated mitochondrial fission (Ong et al. 2015), we isolated mitochondrial and cytoplasmic fractions in each group. The results demonstrate that Drp-1 increased in mitochondria and decreased in the cytoplasm of the virus group. The overexpression of calpastatin partly reversed this CVB3 infection-induced trend (Fig. 3h–j). Overall, these results suggest that calpastatin overexpression suppresses mitochondrial injury caused by excessive mitochondrial fission and is associated with preventing Drp-1 translocation in VMC mice.

### Inhibition of calpain ameliorates CVB3-induced mitochondria-mediated NRCMs’ apoptosis

**Fig. 4:**
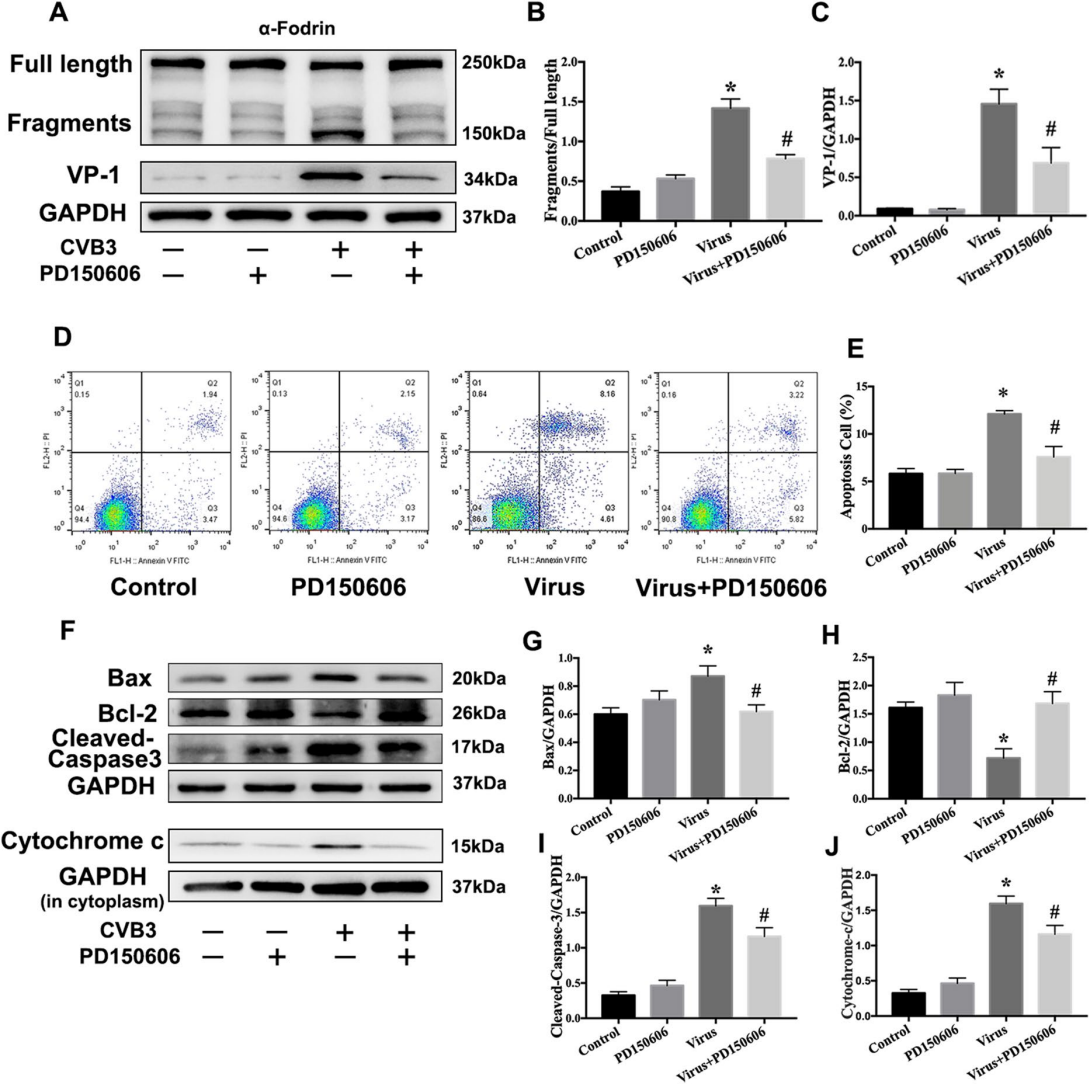
PD150606 ameliorated CVB3-induced mitochondrial-medicated cardiomyocyte apoptosis. **a**–**c** Western bolts were used to evaluate the cleaved-α-fodrin and CVB3 capsid protein VP1 in NRCMs infected with CVB3 with or without PD150606. **d**, **e** Apoptosis was determined by annexin V-FITC/PI staining assay. **f**–**j** Western bolts were used to measure the changes of proteins related to apoptosis, Bax (**g**), Bcl-2 (**h**), cleaved-Caspase-3 (**i**), and cytochrome c in the cytoplasm (**j**). Data are expressed as mean ± SEM. *P < 0.05 vs. control; #*P* < 0.05 vs. virus

Next, we utilized PD150606 (a calpain inhibitor) to further verify our hypothesis in vitro. We examined the calpain activity in CVB3-infected NRCM. We found that PD150606 treatment decreased α-fodrin cleavage induced by CVB3 infection in NRCMs (Fig. 4a, b). PD150606 treatment also inhibited CVB3 replication by reducing the level of VP1 in NRCMs (Fig. 4a, c). The results of the annexin V/PI analysis demonstrated that PD150606 attenuated CVB3-induced cardiomyocyte apoptosis (Fig. 4d, e). We also detected several proteins associated with mitochondria-mediated apoptosis (Fig. 4f–j). We extracted the cytoplasm and found that cytochrome C (cyt-c) was expressed at a higher level during CVB3 infection compared with control group, indicating that CVB3 infection promoted the release of cyt-c into the cytoplasm. The level of cyt-c decreased in the virus + PD150606 group. Furthermore, PD150606 treatment decreased the protein levels of Bax and cleaved-Caspase-3 markedly while increasing the Bcl-2 in NRCMs infected with CVB3. Taken together, these results indicate the anti-apoptotic effect of calpain inhibition during CVB3 infection.

### PD150606 protected mitochondrial function in CVB3-infected NRCMs

**Fig. 5:**
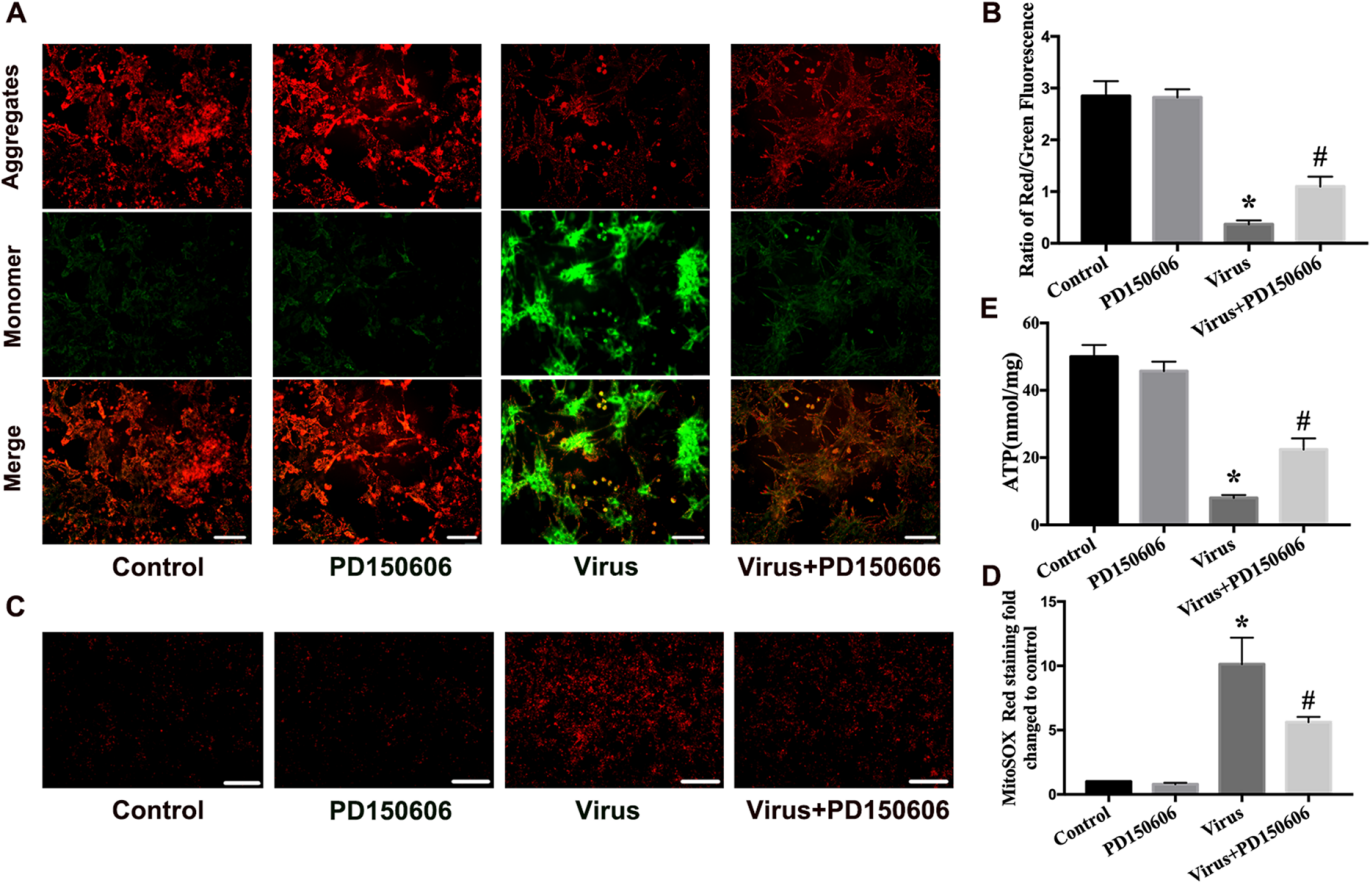
PD150606 protected mitochondrial function in CVB3-infected cardiomyocytes. **a** Mitochondrial membrane potential (MMP) was determined using JC-1 with a fluorescence microscope. The red fluorescence indicates the normal mitochondria potential; the green suggests the damaged mitochondrial potential; scale bar = 50 μm. **b** Ration of red/green fluorescence. **c** The level of matrix superoxide in mitochondria was measured by MitoSox staining; scale bar = 200 μm; **d** MitoSOX red staining fold changes. **e** The ATP level of NRCMs. Data are expressed as mean ± SEM. *P < 0.05 vs. Con; #*P* < 0.05 vs. virus group

To investigate whether PD150606 attenuated CVB3-induced mitochondrial dysfunction, we evaluated mitochondrial function from three different parameters in vitro. JC-1 was used to measure MMP. The control group appeared red. However, there was a drop in MMP after CVB3 infection, as indicated by the stronger green fluorescence and weaker red fluorescence. In comparison with MMP in the virus group, the MMP was higher in the virus + PD150606 group (Fig. 5a, b). MitoSOX staining was used to detect the level of matrix superoxide in mitochondria. After PD150606 pretreatment of CVB3-infected NRCMs, the level of superoxide in mitochondria decreased compared with that of the virus group (Fig. 5c, d). Consistent with the MMP and superoxide results, treatment with PD150606 improved the reduction in ATP synthesis caused by viral infection (Fig. 5e). Taken together, these data suggest that PD150606 intervention protects cardiomyocyte mitochondrial function during CVB3 infection.

### CVB3 infection causes excessive mitochondrial fission in NRCMs

**Fig. 6:**
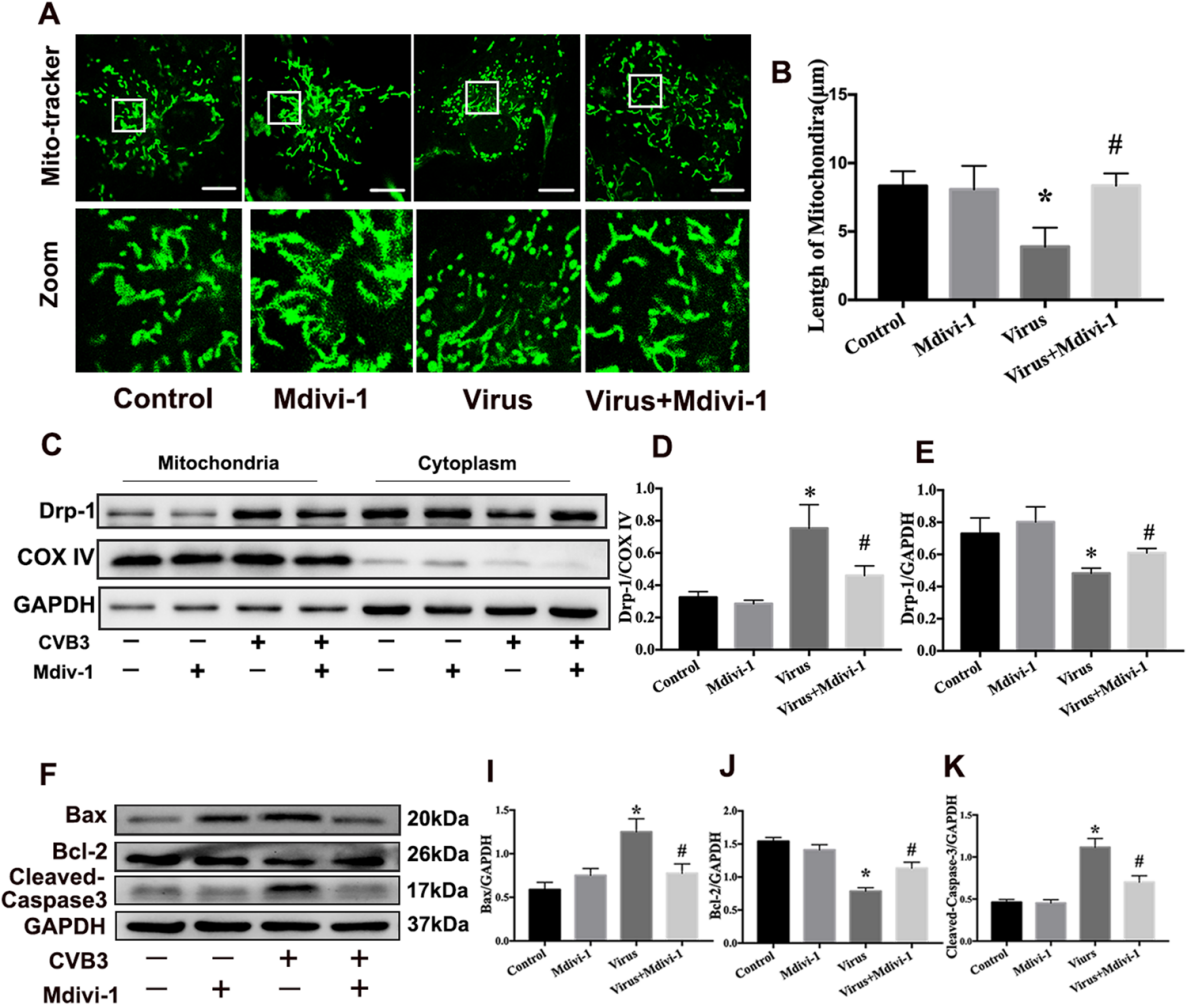
CVB3 infection caused excessive mitochondrial fission in cardiomyocyte, and reduction of mitochondrial fission by Mdivi-1 could alleviate mitochondrial fragmentation and decrease apoptosis caused by CVB3 infection. **a** Mitochondrial morphology was stained with Mitotracker Green in NRCMs infected CVB3 with or without Mdivi-1 (Drp-1 inhibitor); scale bar = 10 μm. **b** Average mitochondrial length was measured by ImageJ software. **c** The mitochondrial proteins and cytoplastic proteins were extracted in NRCMs. Western bolts were used to evaluate Drp-1 expression in mitochondria (**d**) and cytoplasm (**e**), respectively, in NRCMs infected with CVB3 with or without Mdivi-1. **f**–**k** The levels of apoptosis-related proteins were analyzed by western bolts in NRCMs infected with CVB3 with or without Mdivi-1. Bax (**i**), Bcl-2 (**j**), cleaved-Caspase-3 (**k**). Data are expressed as mean ± SEM. *P < 0.05 vs. Con; #*P* < 0.05 vs. virus group

The results of VMC mice showed that CVB3 infection increased the expression of Drp-1 in mitochondria. Therefore, we used Mdivi-1 (Drp-1 inhibitor) to further verify whether CVB3 infection promotes mitochondrial fission and apoptosis through Drp-1. We examined the mitochondrial morphology in CVB3-infected NRCMs using MitoTracker Green staining with confocal microscopy. The mitochondria became fragmented, and the average mitochondrial length was significantly decreased after CVB3 infection. However, mitochondria restored liner shape in virus + Mdivi-1 group (Fig. 6a, b). We also measured the protein levels of Drp-1 in the mitochondria and cytoplasm. The results revealed that Mdivi-1 treatment caused the downregulation of Drp-1 in mitochondria and the upregulation of Drp-1 in the cytoplasm in CVB3-infected NRCMs (Fig. 6c–e). Those results indicated that CVB3 infection increased mitochondrial fission and Mdivi-1 treatment partly restored the mitochondrial morphology in NRCMs infected with CVB3. Furthermore, Mdivi-1 reduced cell apoptosis as demonstrated by decreased levels of Bax and cleaved- Caspase-3 and increased levels of Bcl-2 (Fig. 6f–k). These results indicate that excessive mitochondrial fragmentation mediated by Drp-1 is involved in CVB3 infection and that Drp-1 plays a part in cardiomyocyte apoptosis induced by CVB3 infection.

### PD150606 inhibited mitochondrial fragmentation and regulated Drp-1 translocation

**Fig. 7:**
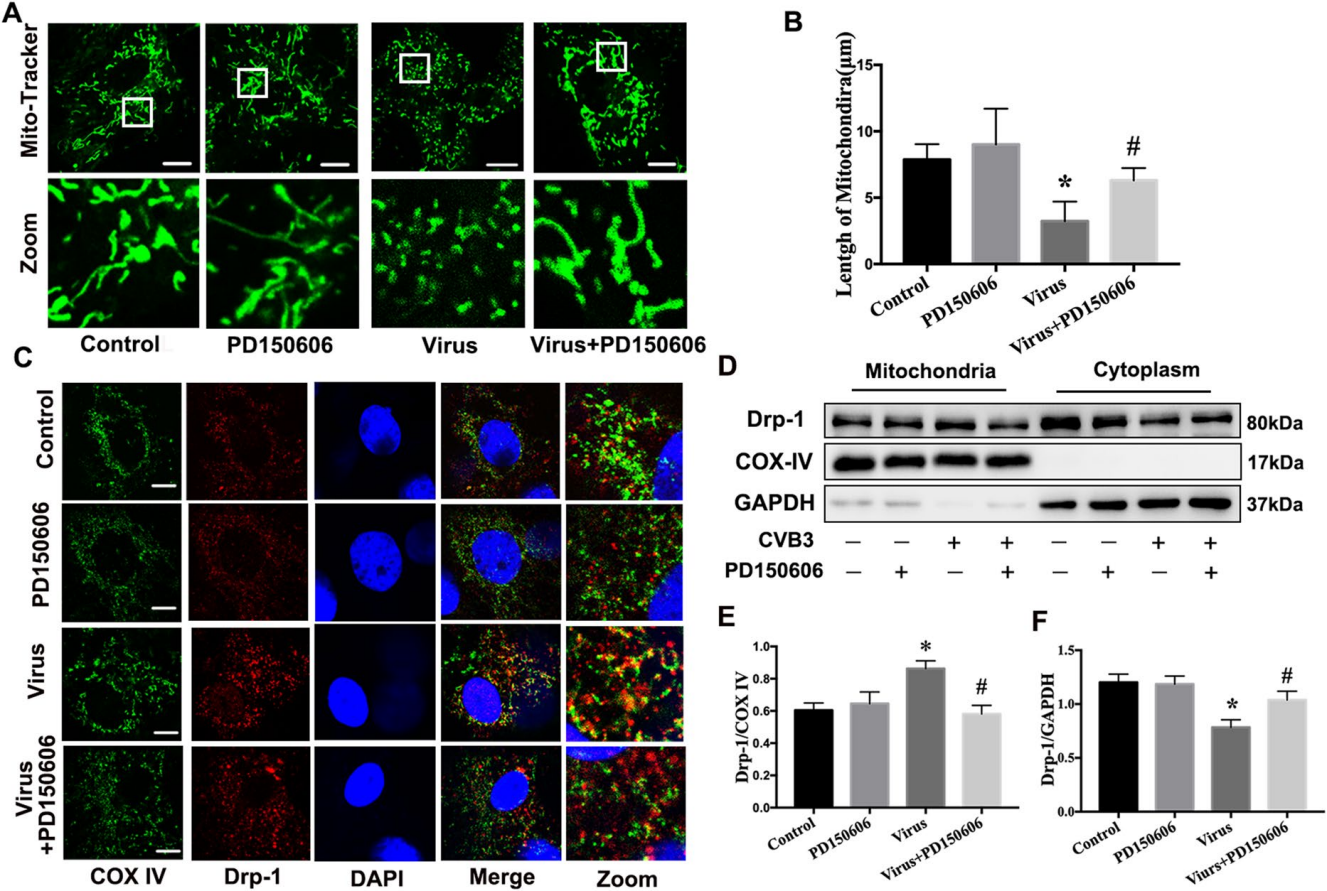
PD150606 inhibited mitochondrial fragmentation and regulated Drp-1 translocation. **a** Mitochondrial morphology was stained with Mitotracker Green in NRCMs infected with CVB3 with or without PD150606. The second panel shows the magnified regions; scale bar = 10 μm. **b** Average mitochondrial length was measured by ImageJ software. **c** The co-staining of mitochondria (green) and Drp-1 (red). In the enlarge panel, the orange fluorescence indicates the co-location of mitochondria and Drp-1; scale bar = 5 μm. **d**–**f** The mitochondrial proteins and cytoplastic proteins were extracted in NRCMs. Western bolts were used to evaluate Drp-1 expression in mitochondria (**e**) and cytoplasm (**f**), respectively, in NRCMs infected with CVB3 with or without PD150606. Data are expressed as mean ± SEM. *P < 0.05 vs. Con; #*P* < 0.05 vs. virus group

As shown in Fig. 7a and b, PD150606 pretreatment restored the structure of mitochondria to some extent in NRCMs infected with CVB3. We used immunofluorescence double staining to observe the location of Drp-1 in NRCMs. PD150606 treatment reduced Drp-1 translocation to the mitochondria in NRCMs infected with CVB3 (Fig. 7c). Consistent with the above results, immunoblotting analysis indicated that the virus + PD150606 group had a lower level of Drp-1 in mitochondria and a higher level of that in the cytoplasm compared with the virus group (Fig. 7d–f). These results suggested that calpain activation caused by CVB3 infection led an increase of Drp-1 mitochondrial localization, thereby causing excessive mitochondrial fission.

### PD150606 inhibited mitochondrial fragmentation by modulating Drp-1 phosphorylation

**Fig. 8:**
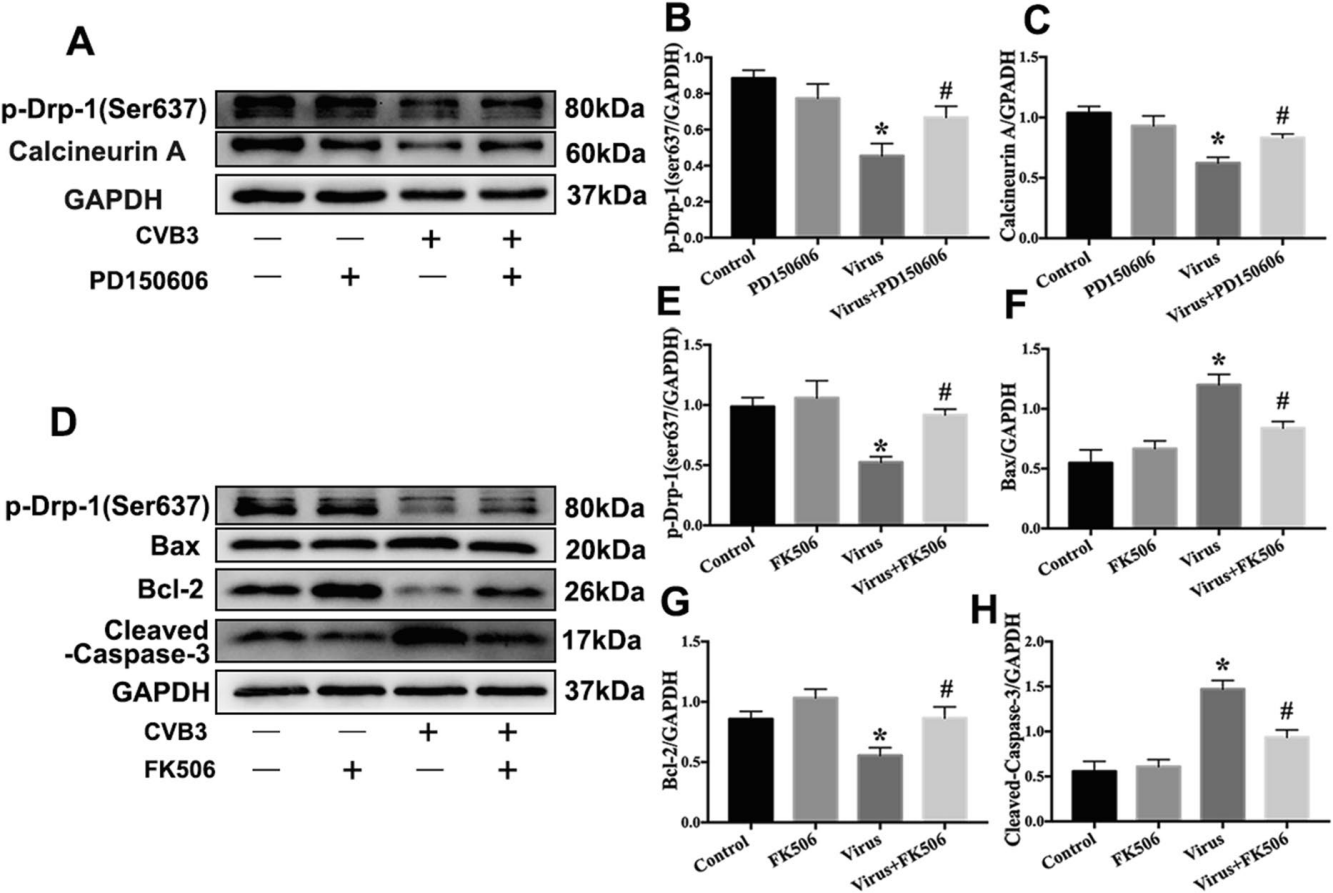
PD150606 inhibited mitochondrial fragmentation by modulating Drp-1 phosphorylation. **a**–**c** The changes of p-Drp-1(Ser637) and calcineurin A in NRCMs infected with CVB3 with or without PD150606. PD150606 treatment inhibited Drp-1 dephosphorylated at the Ser637 site (**b**) and calcineurin activation (**c**) after CVB3 infection. **d**–**h** The changes of p-Drp-1(Ser637) and apoptosis-related proteins in NRCMs infected with CVB3 with or without FK506 (an inhibitor of calcineurin activation). Dephosphorylation of Drp-1 (**e**) was activated by calcineurin activation and contributes to apoptosis (**f**–**h**) in CVB3-infected NRCMs. *P < 0.05 vs. Con; #*P* < 0.05 vs. virus group

The dephosphorylation at Ser637 is a key regulator of mitochondrial fission. Therefore, we measured the phosphorylation levels of Drp-1 at Ser637. It was shown that the phosphorylation levels of Drp-1 at Ser 637 site were reduced after CVB3 infection, while PD150606 treatment promoted phosphorylation at the Ser637 site after CVB3 infection (Fig. 8a, b). It has been reported that activated calpain cleaves calcineurin A and converts it into an active form, which then dephosphorylates Drp-1 at Ser637 (Lee et al. 2016). Therefore, we detected the level of calcineurin A during CVB3 infection. Our results demonstrated that CVB3 infection promoted calcineurin A cleavage, which was inhibited by PD150606 treatment (Fig. 8a, c), implying that calpain activated calcineurin A during CVB3 infection.

Next, we used FK506, an inhibitor of calcineurin activation, to investigate the relationship between calcineurin activation and dephosphorylation of Drp-1 at Ser637. FK506 inhibited dephosphorylation of Drp-1 at Ser637 site induced by CVB3 infection (Fig. 8d, e). Additionally, we observed that CVB3 infection-induced decrease in Bcl-2 expression and enhanced levels of Bax and Caspase-3 cleavage were reversed by FK506 (Fig. 8d, f–h). Taken together, these results indicated that PD150606 inhibits excessive mitochondrial fission by preventing cleavage of calcineurin A, thereby decreasing NRCMs’ apoptosis.

## Discussion

**Fig. 9:**
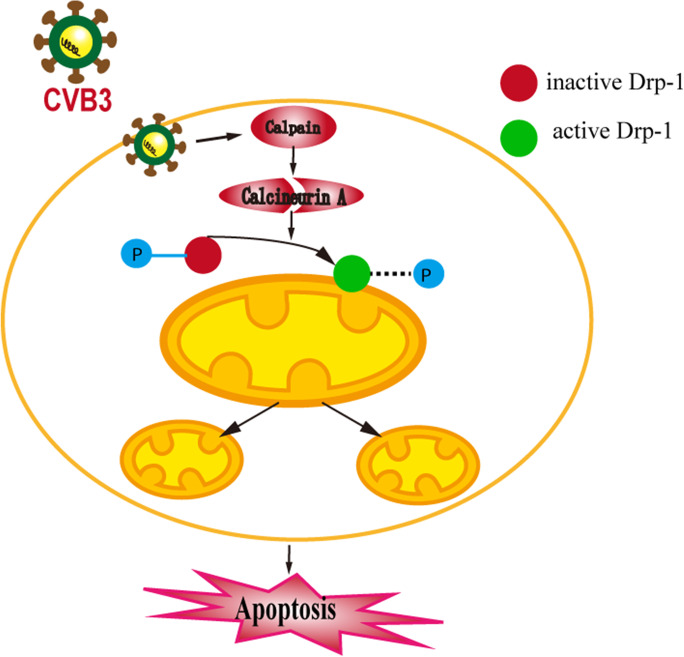
Diagram interpreting the function and mechanism of calpain activation in CVB3-induced acute viral myocarditis. Calpain is activated by CVB3 infection. Activated calpain cleaves calcineurin A and converts it to the active form which could dephosphorylate Drp-1 at Ser637 site. Then, the active Drp-1 translocates from the cytoplasm to mitochondria and triggers excessive mitochondrial fission. Eventually, the balance of mitochondrial dynamics is broken, and apoptosis occurs

The present study demonstrated that calpain was activated in cardiomyocytes infected with CVB3, which was accompanied by mitochondrial dysfunction and excessive mitochondrial fission and increased apoptosis. Nevertheless, inhibition of calpain activity suppressed dephosphorylation of Drp-1 at Ser637 site, prevented Drp-1 translocation, reduced mitochondrial fragmentation, and improved mitochondrial function and decreased cardiomyocyte apoptosis. In brief, we concluded that calpain activation promoted mitochondrial fission by modulating phosphorylation levels of Drp-1 in CVB3-induced myocarditis (Fig. 9).

Calpain activity is involved in many pathological processes in the heart, such as cardiac hypertrophy, inflammatory reaction, fibrosis, and cell apoptosis (Letavernier et al. 2012). Calpastatin, a specific endogenous inhibitor, inhibits calpain activation by binding calpain at its specific domains (Hanna et al. 2008). Moreover, PD150606, a nonpeptide, cell-permeable selective calpain inhibitor, inhibits calpains by binding to the calcium-binding site of the enzyme. Numerous studies have proved that calpain is a promising therapeutic target in many diseases, such as I/R injury (Zu et al. 2020), cardiomyocytes hypertrophy (Li et al. 2020), and renal diseases (Kobayashi-Otsugu et al. 2020). Consist with other researches, our results demonstrated that the inhibition of calpain, both by calpastatin overexpression and PD150606 intervention, attenuated myocardial injury, alleviated cardiac inflammation, improved mitochondrial function, and reduced apoptosis in VMC, implying calpain activation promoted the severity of CVB3-induced myocarditis. However, in recent study, Meng et al. reported that cardiomyocyte-specific capn4-ko mice displayed aggravated symptoms of VMC, manifested by an increase in inflammatory response (Meng et al. 2019). Two transgenic mice differ in the way in inhibiting calpain activity. Myocardial injury caused by inflammatory cells from peripheral tissue or virus released from other infected organs, such as the pancreas, plays important role in the development of VMC (Horwitz et al. 2000; Pinkert et al. 2019). Accordingly, specific inhibition of calpain in cardiomyocytes may not be sufficient to protect the myocardium from the damage caused by peripheral inflammatory cells or other organs. Moreover, other researchers also reported that calpain inhibition protects myocardial injury in VMC (DeBiasi et al. 2001; Li et al. 2016). Therefore, Tg-CAST, systemic overexpression of calpastatin, may exert myocardial protection more through inhibiting damage caused by peripheral tissues, which needs our more research to find out the underlying mechanisms.

Mitochondria are known not only for their role as a powerhouse that supplies energy for cells but also for their role in cell apoptosis. To induce apoptosis, several changes need to occur in mitochondria, such as mitochondrial fragmentation, cristae remodeling, and mitochondrial outer membrane permeabilization. These changes promote the release of cytochrome c and other proapoptotic factors, ultimately leading to caspase activation and cell death (Kasahara and Scorrano 2014). Balance of mitochondrial dynamic is critical to the maintenance of mitochondrial function. However, viruses disturb this balance to ensure their survival and escape from innate immunity, thereby facilitating infection (Khan et al. 2015; Mukherjee et al. 2018). Mitochondrial fragmentation and activation of intrinsic apoptotic pathway are observed during rotaviruses (RV) and porcine reproductive and respiratory syndrome virus infections (Mukherjee et al. 2018; Pujhari and Zakhartchouk 2016). Therefore, mitochondrial dynamics plays critical roles in apoptosis induced by viral infection. In agreement with previous studies, our present study found that CVB3 infection changed the mitochondrial morphology and inhibition of excessive mitochondrial fission reduced NRCMs’ apoptosis.

In the present study, we detected the mRNA levels of the major molecules regulating mitochondrial dynamic. We found increased expression of Drp-1 and decreased expression of Mfn-2 and Opa-1. Studies have demonstrated that disrupted mitochondrial homeostasis caused by Drp-1 is related to many vascular disorders, such as endothelial dysfunctions, cardiac hypertrophy, heart failure, and so on, making Drp-1 as a potential therapeutic target in cardiovascular diseases (Jin et al. 2021; Sharp 2015). Moreover, pharmacological inhibition of Drp-1 attenuates mitochondrial fragmentation and myocardial injury in myocarditis induced by CVB3 (Lin et al. 2017). Therefore, we focused on the effect of CVB3 on Drp-1. Drp-1, a protein that is a member of the dynamin GTPase superfamily, is essential for mitochondrial fission. Inactive Drp-1 is present in the cytoplasm. When activated, it translocates to the OMM and triggers mitochondrial fission. In the present study, we indicated that CVB3 infection triggered mitochondrial fission, which was accompanied by an accumulation of Drp-1 located on OMM. However, the underlying regulatory mechanisms have not yet been elucidated.

The roles of calpain in regulating mitochondrial function have been elaborated in many diseases, such as dilated heart failure(Cao et al. 2019) and diabetic hearts (Zheng et al. 2020). Consistent with the above studies, we proved that calpain activity was involved in the modulation of mitochondrial function in VMC, as reflected by the decreases in ATP content, MMP collapse, and release of proapoptotic factors in mitochondria. However, using calpain inhibitors or transgenic mice overexpressing calpastatin resulted in improved mitochondrial function.

Furthermore, we explored the mechanisms of calpain involvement in mitochondrial function in VMC. Recently, studies have revealed the relationship between calpain and mitochondrial dynamics. Calpain activation leads to the degradation of mitochondrial fusion protein, Mfn-2 (Xue et al. 2017). Endothelial cells exposed to hyperglycemia have increased mitochondrial fragmentation, which is associated with increased calpain activity (Ong et al. 2019). In neuroblastoma cells treated with H_2_O_2_, calpastatin is able to alleviate disturbances in mitochondrial dynamics by decreasing mitochondrial fission protein (Fis-1 and Drp-1) levels (Tangmansakulchai et al. 2016). Our present research revealed that both PD150606 and calpastatin overexpression successfully inhibited Drp-1 migration from the cytoplasm to OMM and restored the morphology of the mitochondria in the VMC model. Post-translational modification, such as phosphorylation, controls the activity of Drp-1, including Ser637 (dephosphorylation) and Ser616 (phosphorylation) (Ong et al. 2015). We found that Ser637 of Drp-1 was dephosphorylated during CVB3 infection. It has been reported that Ser637 in Drp-1 is dephosphorylated by calcineurin both in neurons and cardiomyocyte (Huang et al. 2019; Lee et al. 2016; Pennanen et al. 2014). As a calcium and calmodulin-dependent protein phosphatase, calcineurin could be cleaved and activated by calpain, which increases phosphatase activity of calcineurin (Wu et al. 2007; Zhao et al. 2016). Calpain/calcineurin pathway also participates in pyroptosis (Zu et al. 2020) and neuron injury (Lei et al. 2018). Therefore, we examined whether the calpain/calcineurin pathway is involved in VMC. We found that calpain could cleave calcineurin A during CVB3 infection. And inhibition of calcineurin activity by FK506 decreased the levels of dephosphorylation of Drp-1 at Ser637 and reduced NRCMs apoptosis. Our present study indicates that calpain activation caused by CVB3 infection promoted Drp-1 dephosphorylation at Ser637 site through cleaving calcineurin A, which initiates mitochondrial fission.

In summary, our findings demonstrate calpain activation induced by CVB3 infection promoted mitochondrial fission via modulating Drp-1 phosphorylation at Ser637 site and then resulted in myocardial apoptosis. Our study illustrates the role of calpain activity in the pathogenesis of VMC and provides new evidence for finding therapeutic targets. However, further studies by inhibiting calpain activity in mitochondria will shed light on the relationship between calpain and mitochondria, which is the focus of our next research.

## Data Availability

The data are available on request from the corresponding author.
